# Reports of Negative Interactions with Healthcare Providers among Transgender, Nonbinary, and Gender-Expansive People assigned Female at Birth in the United States: Results from an Online, Cross-Sectional Survey

**DOI:** 10.3390/ijerph20116007

**Published:** 2023-05-31

**Authors:** Elizabeth M. Inman, Juno Obedin-Maliver, Sachiko Ragosta, Jen Hastings, Jasmine Berry, Mitchell R. Lunn, Annesa Flentje, Matthew R. Capriotti, Micah E. Lubensky, Ari Stoeffler, Zubin Dastur, Heidi Moseson

**Affiliations:** 1 Department of Psychology, Stony Brook University, 100 Nicolls Road, Stony Brook, NY 11794, USA; 2 The PRIDE Study/PRIDEnet, Stanford University School of Medicine, 3180 Porter Drive, Palo Alto, CA 94304, USA; 3 Department of Obstetrics and Gynecology, Stanford University School of Medicine, 300 Pasteur Drive, Stanford, CA 94305, USA; 4 Department of Epidemiology and Population Health, Stanford University School of Medicine, 300 Pasteur Drive, Stanford, CA 94305, USA; 5 Ibis Reproductive Health, 1736 Franklin, Oakland, CA 94612, USA; 6 Department of Family and Community Medicine, University of California, 995 Portrero Ave, San Francisco, CA 94410, USA; 7 Division of Nephrology, Department of Medicine, Stanford University School of Medicine, 300 Pasteur Drive, Stanford, CA 94305, USA; 8 Department of Community Health Systems, University of California, 2 Koret Way, San Francisco, CA 94143, USA; 9 Alliance Health Project, Department of Psychiatry, University of California, 1930 Market Street, San Francisco, CA 94102, USA; 10 Department of Psychology, San José State University, 1 Washington Square, San Jose, CA 94192, USA

**Keywords:** gender-affirming care, nonbinary, patient care, quality of care, stigma, transgender

## Abstract

Over one million people in the United States are transgender, nonbinary, or gender expansive (TGE). TGE individuals, particularly those who have pursued gender-affirming care, often need to disclose their identities in the process of seeking healthcare. Unfortunately, TGE individuals often report negative experiences with healthcare providers (HCPs). We conducted a cross-sectional online survey of 1684 TGE people assigned female or intersex at birth in the United States to evaluate the quality of their healthcare experiences. Most respondents (70.1%, *n* = 1180) reported at least one negative interaction with an HCP in the past year, ranging from an unsolicited harmful opinion about gender identity to physical attacks and abuse. In an adjusted logistic regression model, those who had pursued gender-affirming medical care (51.9% of the sample, *n* = 874) had 8.1 times the odds (95% CI: 4.1–17.1) of reporting any negative interaction with an HCP in the past year, compared to those who had not pursued gender-affirming care, and tended to report a higher number of such negative interactions. These findings suggest that HCPs are failing to create safe, high-quality care interactions for TGE populations. Improving care quality and reducing bias is crucial for improving the health and well-being of TGE people.

## 1. Introduction

Despite increasing public awareness of the diversity of gender identity, transgender, nonbinary, and gender expansive (TGE) people continue to experience stigmatization and discrimination broadly, including in healthcare settings [1]. In the United States, an estimated 1.6% of all adults and 5.1% of young adults aged 18–30 years old identify as transgender or nonbinary—meaning that their gender identity is different from what is commonly assumed based on the sex assigned to them at birth [2,3]. Although almost half of all US adults knew at least one TGE person in 2022, the number of survey respondents who believe that gender identity is determined by sex assigned at birth increased from 2021 [4]. Additionally, legislation restricting the rights of TGE people continues to increase each year. In 2022, over 150 bills were proposed in state legislatures to allow refusal of service to TGE individuals due to religious beliefs, to restrict TGE individuals to using public bathrooms corresponding to their sex assigned at birth rather than their gender, or to limit TGE individuals’ access to gender-affirming care [5]. These events foment transphobia—hate and discrimination against transgender people. Discriminatory laws and transphobia worsen the quality of life and mental health outcomes among TGE individuals [6].

Access to healthcare is a protective factor for TGE individuals’ well-being. For many TGE people, though certainly not all, gender-affirming care (e.g., puberty blockers, hormone therapy, gender-affirming surgery) is an important part of transitioning—the process of medically and/or surgically aligning oneself with one’s gender identity rather than one’s sex assigned at birth [7]. Accessing gender-affirming care, particularly during adolescence, is associated with lower odds of stress, depression, and suicidal ideation [8,9,10,11]. Based on this evidence, five states have recently passed legislation to ensure access to gender-affirming care for TGE people [12]. Conversely, restricting access to care and encouraging TGE people to present as the gender assumed for their sex assigned at birth is associated with increased levels of anxiety, depression, and suicidal ideation [13,14]. Healthcare access and engagement also increase necessary preventive care (e.g., annual physical exams) and appropriate prenatal care among TGE individuals who choose to carry a pregnancy [7,15]. Access to care alone is not enough to improve TGE patient outcomes; the quality of care received is vital.

Quality of care may be impacted by various factors, including interactions with healthcare providers (HCPs) [16]. Unfortunately, TGE patients classify most experiences with HCPs as negative, either due to bias or a lack of education among HCPs [17,18]. Negative interactions with healthcare providers are associated with lower access to preventive and gender-affirming medical care and with maladaptive coping behaviors, including abuse of drugs and alcohol [19,20,21]. These negative healthcare experiences may be more common among patients who are out about their gender identity and who engage in gender-affirming care [22,23]. Although some TGE patients may not want to share their gender identity with HCPs, medical histories that list current medications or previous surgeries can unintentionally disclose people’s identities/experiences or “out” patients and bias HCP behavior [24,25]. Similarly, pursuing gender-affirming care inherently requires frequent disclosure of a TGE identity and an increase in the frequency of interactions with HCPs, creating more opportunities for negative experiences and biased interactions. Given the link between care quality and TGE well-being, it is important to assess the quality of TGE healthcare experiences and identify areas of improvement.

We sought to evaluate the quality of TGE care experiences from the patient perspective. Within a large cross-sectional online survey study of TGE people assigned female sex at birth in the United States, we collected data on past-year interactions with HCPs. We examined these experiences for the entire sample and examined potential associations with care history and sociodemographic characteristics to identify potential areas of improvement and intervention.

## 2. Methods

### 2.1. Study Population

We recruited participants between May and September 2019 for an online survey about a range of sexual and reproductive health experiences. To join the study, participants had to be at least 18 years old, able to read and understand English, live in the United States, have been assigned female or intersex sex at birth, and identify as TGE. We recruited from the general US population using study advertisements posted on social media (e.g., Reddit, Instagram, Twitter) and shared through listservs and at in-person events (e.g., academic conferences, community Pride events). Additionally, we recruited participants through an ongoing, online, prospective, longitudinal, cohort study of sexual and gender minority adults: The Population Research in Identities and Disparities for Equality (PRIDE) Study [26,27].

### 2.2. Procedures

Interested participants were directed to an online screener questionnaire. If eligible, they completed an informed consent process and continued to the online, self-administered survey hosted by Qualtrics (Qualtrics, LLC; Provo, UT). The survey leveraged customizable language and skip logic to allow participants to use their preferred language for sexual and reproductive health terms in order to minimize dysphoria. Detailed descriptions of survey development and results of customizable survey content have been previously published [28,29]. Participants were entered into raffles to win one of 100 $50 electronic gift cards. The study was approved by the Institutional Review Boards at the University of California, San Francisco, Stanford University School of Medicine, and now is held in addition by the WCG IRB for ongoing analysis. Additionally, the study was reviewed and approved by The PRIDE Study Research Advisory Committee and Participant Advisory Committee.

### 2.3. Measures

The primary outcome of interest—negative healthcare interactions—was assessed with a set of healthcare experiences drawn from the 2015 US Transgender Survey [13] and from The PRIDE Study cohort. Participants responded to a checklist of 16 potential negative events (e.g., “I had to teach my doctor/healthcare provider about transgender and gender-expansive people so that I could get appropriate care”) and indicated which, if any, events they had experienced in the past year. Participants could also indicate additional negative events that were not listed in an open-text response. The full list of experiences is represented in Table 2.

Participants completed a nine-item checklist summarizing their experiences with gender-affirming care. Participants who indicated that they had received gender-affirming hormone therapy, puberty blockers, gender-affirming genital surgery, other gender-affirming surgery (e.g., chest reduction or reconstruction), or any combination of these interventions were identified as having a history of gender-affirming care. Participants who indicated that the question was irrelevant, that they had not sought any gender-affirming care, or that they had sought gender-affirming care but could not access it, were identified as not having any history of gender-affirming care.

Due to the research suggesting more negative experiences among patients who have disclosed a TGE gender identity, we asked participants to indicate the percentage of their HCPs who they thought were aware of their TGE identity. Participants estimated the proportion of their HCPs who knew about their gender identity on a scale of 0 (out to 0% of providers) to 10 (out to 100% of providers).

Participants reported sociodemographic characteristics including their current gender identity, their sex assigned at birth, race and ethnicity, age, income, education level, and health insurance status. Current gender identity was assessed with the question “What best describes your current gender identity at this time?” Participants could select all options that apply from a list, including an open-text response. Sex assigned at birth was assessed with the question “What sex were you assigned at birth, for example on your original birth certificate?” Participants could respond with a female, male, or open-text response option. Additional details on the wording and format of these questions have been previously published [28].

### 2.4. Statistical Analysis

We conducted all analyses using R [30]. To examine the distribution of healthcare experiences, we calculated overall frequencies and ranges for each healthcare experience as well as stratified by history of gender-affirming care. Because the past-year healthcare experiences outcome could be conceptualized as a dichotomous variable or a count variable, we used both logistic regression and binomial regression to evaluate the association between engaging in gender-affirming care and negative experiences. In both models, we adjusted for participant age (continuous), race and ethnicity (categorical), census region (categorical), education level (categorical), and level of outness to healthcare providers (categorical). We also tested the level of outness as a potential modifier of the link between engaging in gender-affirming care and negative experiences.

## 3. Results

### 3.1. Participant Demographic Characteristics

Overall, 3110 respondents completed the survey, of whom 1684 were TGE and completed the survey modules of interest for this analysis. These participants had a median age of 27.1 years old (IQR = 10.1) and a median annual income of $ 52,000 (Table 1). Participants reported high levels of education with many having earned a college degree (*n* = 514, 30.5%) or a graduate/professional degree (*n* = 408, 24.2%). A majority of the sample (*n* = 1464, 86.9%) identified as White. Most (*n* = 1524, 90.5%) had health insurance coverage. Participants identified with a range of gender identities including nonbinary (*n* = 861, 51.1%), transgender man (*n* = 661, 39.2%), and genderqueer (*n* = 651, 38.6%).

### 3.2. Gender-Affirming Care

Approximately half (*n* = 874, 51.9%) of survey respondents had engaged in some form of gender-affirming care (i.e., puberty blockers, hormone therapy, surgery). Of these participants, most reported engaging in gender-affirming hormone therapy (*n* = 802, 91.8%), followed by having had a gender-affirming surgery (*n* = 636, 72.8%) and having used puberty blockers (*n* = 17, 1.9%) (Table 1).

### 3.3. Gender-Identity Outness to Healthcare Providers

Participants reported what percentage of their HCPs were aware of their gender identity. These responses were concentrated at the extremes, with 20.1% (*n* = 338) of the sample indicating that they were not out to any of their HCPs, and 21.1% (*n* = 356) of the sample indicating that they were out to 100% of their HCPs.

### 3.4. Negative Healthcare Experiences

Overall, 70.1% (*n* = 1180) of participants reported one or more of the measured negative healthcare experiences in the past year (Table 2). The number of negative experiences ranged from 0 to 14 (median = 2.0, IQR = 4.0). The most frequently reported experiences included being negatively affected by an HCP’s opinions about gender identity and/or sexual orientation (*n* = 955, 56.7%) and having to educate their HCP about gender identity (*n* = 565, 33.5%) or sexual orientation (*n* = 429, 25.5%) to receive proper medical care. Other reported experiences included receiving varying levels of care (*n* = 472, 28.0%), being referred to specialists who are not gender-affirming or inclusive (*n* = 342, 20.3%), and being asked inappropriate questions about gender identity (*n* = 336, 19.9%). Fewer than one in five (*n* = 283, 17%) survey respondents reported no negative interactions with HCPs in the past year.

When examined through the lens of gender-affirming care, overall negative past-year healthcare experiences were particularly high among respondents who had received gender-affirming care (*n* = 691, 79.1%) compared to respondents who had not received gender-affirming care (*n* = 489, 60.6%). In 15 of the 16 surveyed experiences, frequency scores were higher for participants with a history of gender-affirming care. These differences were statistically significant in 13 of the 16 surveyed experiences (Table 2). Only 6.9% (*n* = 60) of respondents who had received gender-affirming care reported no negative interactions with HCPs in the past year.

In an adjusted logistic regression model, a history of gender-affirming care was associated with increased odds of reporting any negative healthcare interaction in the past year by 813% (OR = 8.1, 95% CI = 4.1–17.1; Table 3). Outness to providers modified the association between a history of gender-affirming care and any reported negative experience (B = −0.17, *p* < 0.001) such that the level of outness to HCPs was associated with greater odds of reporting any negative experience among participants who did not have a history of gender-affirming care, as compared to participants with a history of gender-affirming care.

In a similarly adjusted binomial regression model with the *number* of reported negative experiences in the last year as a count outcome, gender-affirming care was associated with an increase in the incidence rate of negative experiences by 309% (OR = 3.1, 95% CI = 2.4–3.9; Table 4). Outness to providers similarly modified the association between a history of gender-affirming care and the number of negative experiences (B = −0.09, *p* < 0.001) such that the level of outness to HCPs was associated with a higher incidence rate of negative experiences among participants who did not have a history of gender-affirming care, as compared to those with a history of gender-affirming care.

## 4. Discussion

The purpose of this study was to examine the healthcare experiences of TGE people in the United States. In this sample of 1684 survey respondents, we found that 70.1% of participants had experienced at least one negative interaction with an HCP in the past year, with 848 (50.3%) experiencing more than one, and that these experiences are particularly frequent among TGE individuals who had received gender-affirming care. There are several important implications of these findings for TGE health outcomes.

We found similar levels of negative healthcare experiences among TGE patients as prior studies, suggesting stagnant levels of HCP awareness and training among HCPs [13,31]. Negative past-year healthcare experiences were particularly high among participants who had engaged in gender-affirming medical care, and history of gender-affirming care was strongly associated with increased odds of reporting any of these experiences. Among those without a history of gender-affirming care, higher levels of outness to HCPs about one’s gender identity were associated with increased odds of reporting negative experiences. Quantitative research and personal narratives have reported on the link between gender-affirming care and more negative interactions with HCPs [22,23,24,25]. This study confirms those personal experiences among a large sample.

Recent work has identified potential factors that may help explain this phenomenon. Indirect disclosure of a TGE identity (e.g., identity disclosure through medical records) may be interpreted more negatively than direct disclosure (e.g., in a conversation with the HCP) among HCPs who already hold transphobic views [32]. The effect of direct vs. indirect disclosure may explain the interaction between the history of gender-affirming care and outness. Among participants who had received gender-affirming care, higher levels of outness to providers attenuated the link between gender-affirming care and experience of negative interactions. It is possible that participants who received gender-affirming care but were not out to providers were being “outed” by their medical records, and that this indirect disclosure could bias HCP attitudes and create more negative encounters. Toward the goal of improving patient experiences, HCPs should be aware of this implicit bias.

This sample included a range of gender identities, highlighting important intragroup differences. Nonbinary individuals who want gender-affirming care experience lower access to care, compared to binary transgender individuals [33]. Nonbinary individuals experience unique barriers to care, including intake forms that only include binary gender options and confusion from HCPs about the nuance of seeking gender-affirming care with a nonbinary gender identity [34]. More broadly, attitudes toward nonbinary or gender-expansive individuals may be more negative than attitudes toward binary transgender individuals [35]. In this sample, nonbinary and genderqueer identities were more frequently reported among those without a history of gender-affirming care. The interaction between gender-affirming care and outness may reflect these barriers or negative social attitudes toward nonbinary individuals, including bias from HCPs specific to nonbinary identity.

Although we have limited information on participants’ HCPs, the gender identity of the HCP may impact patient care for TGE populations, as cisgender men, including cisgender men who work as HCPs, exhibit higher levels of transphobia than cisgender women [36]. Importantly, previous research [32,35] concludes transphobic attitudes, not patient behavior or identity, are driving the link between receiving gender-affirming care and negative interactions. Future research should seek to capture HCP perspectives on their competence in TGE treatment, particularly in comparison to patient perspectives.

These findings are concerning, but they should not be misinterpreted to suggest that gender-affirming care itself causes negative interactions between HCPs and TGE patients. Individuals pursuing gender-affirming care likely have more frequent interactions with HCPs in general and therefore have more chances for a negative experience and a greater number of interactions to consider when asked about those experiences. These negative interactions are not the patient’s fault but are the result of bias at an individual and policy level within the medical system. When care within the medical system is inaccessible or inadequate, TGE individuals may self-manage their care outside of the system [37,38]. Improving care is necessary so that TGE individuals can have the option of obtaining their gender-affirming care alongside a trained HCP in an affirming, affordable, and accessible environment. There are several published guidelines for improving and standardizing care for TGE patients which HCPs can use as resources, including the World Professional Association for Transgender Health (WPATH) Standards of Care and The Endocrine Society’s Clinical Practice Guideline on gender dysphoria/gender incongruence [39,40].

There are a few important limitations to this study. First, the cross-sectional survey format limits our ability to make conclusions about causality. However, our regression models evaluated exposures of past-year experiences that were established prior to the past year in a temporally appropriate way, which supports testing these exposures again in a subsequent longitudinal study. Second, we are unable to test for differences between HCPs who provide gender-affirming care specifically compared to HCPs who provide other kinds of care. It is likely that HCPs who are trained specifically in gender-affirming care engage in fewer negative interactions. Finally, this sample is majority White, highly educated, and highly insured, which limits our ability to examine how healthcare experiences might differ among participants among people with different ethnoracial and socioeconomic identities. Given previous work on healthcare experiences among TGE people of color and TGE people on public insurance [22,41,42], the levels of stigma and discrimination reported by our survey respondents are likely *lower* than the levels of stigma and discrimination faced by the broader TGE population. Similarly, our sample only included participants who were assigned female or intersex at birth. The broader TGE population would be likely impacted by transmisogyny (i.e., bias against transgender women) in addition to TGE stigma [23,43].

## 5. Conclusions

The current study found that TGE people continue to experience negative, biased interactions in healthcare settings, particularly if they have accessed gender-affirming care. HCPs must adjust their practices to meet published guidelines and to provide their patients with respectful, quality care that affirms gender diversity. We recommend increasing and improving training about TGE healthcare in medical education settings [44,45]. Stigma reduction interventions and increasing diversity in HCP populations may mitigate bias, although more research is needed in these areas [46,47]. Inclusive data collection options in electronic medical record systems and comprehensive insurance coverage for gender-affirming care would also improve patient experiences [48,49]. Importantly, reducing transphobia will improve care more effectively than brief training or interventions, therefore larger systemic changes are needed to shift public attitudes [50]. As aggressive anti-trans legislation and bills are being proposed to curtail transgender rights around the country, emboldening and validating transphobic behavior, we must more proactively work to restore legal protections of TGE people and better equip HCPs to adequately address TGE healthcare needs so that TGE people do not need to compromise their healthcare needs or conceal their identities to receive quality, accurate care.

## Figures and Tables

**Table 1:** Respondent sociodemographic characteristics among an online sample of transgender, nonbinary, and gender-expansive people in the United States (*n* = 1684).

	Full Sample (*n* = 1684)	Any GA ^a^ Care (*n* = 874)	No GA Care (*n* = 807)
Age *Mdn* (*IQR*)	27.1 (10.1)	28.0 (9.9)	26.5 (10.2)
Income *Mdn* (*IQR*)	52,000 (59,000)	50,000 (56,000)	55,000 (58,050)
Gender identity ^b^ *n* (%)			
Nonbinary	861 (51.1)	350 (40.0)	510 (63.2)
Transgender man	661 (39.2)	572 (65.4)	87 (10.8)
Genderqueer	651 (38.6)	226 (25.9)	425 (52.7)
Man	293 (17.4)	274 (31.4)	18 (2.2)
Agender	226 (13.4)	92 (10.5)	134 (16.6)
Woman	201 (11.9)	16 (1.8)	185 (22.9)
Cisgender woman	94 (5.6)	3 (0.3)	91 (11.3)
Two-spirit	25 (1.5)	13 (1.5)	11 (1.4)
Transgender woman	2 (0.1)	0 (0)	1 (0.1)
Cisgender man	1 (0.06)	0 (0)	1 (0.1)
Additional gender	194 (11.5)	90 (10.3)	104 (12.9)
Prefer not to say	2 (0.1)	0 (0)	2 (0.2)
Intersex *n* (%)	107 (6.3)	69 (7.9)	38 (4.7)
Race ^b^ *n* (%)			
White	1464 (86.9)	774 (88.6)	690 (85.5)
Hispanic/Latinx	101 (6.0)	53 (6.1)	48 (5.9)
Black/African American	67 (4.0)	31 (3.5)	36 (4.5)
American Indian/Alaskan Native	42 (2.5)	25 (2.9)	17 (2.1)
East Asian	41 (2.4)	21 (2.4)	20 (2.5)
Southeast Asian	25 (1.5)	6 (0.7)	19 (2.4)
Middle Eastern/North African	24 (1.4)	17 (1.9)	7 (0.9)
South Asian	19 (1.1)	11 (1.3)	8 (1.0)
Native Hawaiian/Pacific Islander	5 (0.3)	3 (0.3)	2 (0.2)
Central Asian	0 (0)	0 (0)	0 (0.0)
Unknown	12 (0.7)	3 (0.3)	9 (1.1)
None	4 (0.2)	1 (0.1)	3 (0.4)
Education level *n* (%)			
Some high school or less	23 (1.4)	11 (1.3)	12 (1.5)
High school degree/GED	118 (7.0)	53 (6.1)	65 (8.1)
Some trade school	9 (0.5)	8 (0.9)	1 (0.1)
Trade school degree	22 (1.3)	12 (1.4)	10 (1.2)
Some college	378 (22.4)	203 (23.2)	175 (21.7)
College degree	514 (30.5)	260 (29.7)	254 (31.5)
Some graduate/professional school	125 (7.4)	65 (7.4)	60 (7.4)
Graduate/professional degree	408 (24.2)	221 (25.3)	187 (23.2)
Health insurance coverage *n* (%)	1524 (90.5)	801 (91.6)	723 (89.6)
Parent status *n* (%)	197 (11.7)	107 (12.2)	90 (11.2)
Gender-affirming care *n* (%)			
Gender-affirming hormones	802 (47.6)	802 (91.8)	NA
Gender-affirming surgery (non-genital)	524 (31.1)	524 (60.0)	NA
Gender-affirming genital surgery	115 (6.8)	115 (13.2)	NA
Puberty blockers	17 (1.0)	17 (1.9)	NA
Two types of GA care	430 (25.5)	430 (49.2)	NA
Three types of GA care	74 (4.4)	74 (8.5)	NA
All four types of GA care	2 (0.1)	2 (0.1)	NA

^a^ Abbreviation: Gender-affirming (GA); ^b^ Participants could select more than one option, percentages may be greater than 100%.

**Table 2:** Negative healthcare experiences, overall and by gender-affirming care history, among an online sample of transgender, nonbinary, and gender-expansive people in the United States (*n* = 1684).

Experience	Full Sample (*n* = 1684)	Any GA Care (*n* = 874)	No GA Care (*n* = 807)	Difference between Groups (Logistic Regression)
	*n* (%)	*n* (%)	*n* (%)	*p*
Negatively affected by HCP’s opinions about gender or sexual identity	955 (56.7)	578 (66.1)	377 (46.7)	<0.001
Had to educate HCP about TGE people to receive care	565 (33.5)	432 (49.4)	133 (16.5)	<0.001
Received varying levels of care from different providers	472 (28.0)	311 (35.6)	161 (20.0)	<0.001
Had to educate HCP about LGBQ people to receive care	429 (25.5)	270 (30.9)	159 (19.7)	<0.001
Referred to unaffirming specialist (TGE identity)	342 (20.3)	243 (27.8)	99 (12.3)	<0.001
HCP asked inappropriate questions about TGE identity	336 (19.9)	284 (32.5)	52 (6.4)	<0.001
HCP asked inappropriate questions about LGBQ identity	197 (11.7)	131 (15.0)	66 (8.2)	< 0.001
Referred to unaffirming specialist (LGBQ identity)	172 (10.2)	107 (12.2)	65 (8.1)	<0.001
Harsh speech from HCP	138 (8.2)	86 (9.8)	52 (6.4)	0.01
Provider refused TGE-related care	118 (7.0)	97 (11.1)	21 (2.6)	<0.001
Provider refused general care	91 (5.4)	66 (7.6)	25 (3.1)	<0.001
Verbal harassment in healthcare setting	55 (3.3)	37 (4.2)	18 (2.2)	<0.001
Physical abuse from HCP	46 (2.7)	27 (3.1)	19 (2.4)	0.36
Provider refused LGBQ-related care	31 (1.8)	27 (3.1)	4 (0.5)	<0.001
Unwanted sexual contact in healthcare setting	29 (1.7)	18 (2.1)	11 (1.4)	0.23
Physical attack in healthcare setting	3 (0.2)	1 (0.1)	2 (0.2)	0.53
Not listed (e.g., misgendering, deadnaming)	168 (10.0)	82 (9.4)	86 (10.7)	0.28
None	283 (16.8)	60 (6.9)	223 (27.6)	<0.001

Note. Participants could select more than one negative experience, percentages may be greater than 100%; Abbreviations: GA = Gender-affirming care; HCP = health care provider; LGBQ = lesbian, gay, bisexual, queer; TGE = transgender, nonbinary, gender-expansive; Definition: Misgendering = referring to a person using gender-specific words that do not align with their gender identity; Deadnaming = referring to a TGE person by a name they used prior to transitioning.

**Table 3:** Logistic regression model output for the association between receipt of gender-affirming care and report of any negative healthcare experience, adjusted for sociodemographic characteristics and outness to healthcare providers (*n* = 1383).

	B	SE	*z*	*p*	OR	95%CI
Intercept	−0.61	0.33	−1.86	0.06	0.54	0.29–1.03
Age	−0.003	0.01	−0.35	0.73	1.00	0.98–1.01
Race	0.04	0.19	0.18	0.86	1.03	0.72–1.52
Census region	−0.02	0.06	−0.50	0.62	0.97	0.87–1.09
Education level	0.50	0.12	4.30	< 0.001	**1.64**	1.31–2.06
Outness	0.06	0.03	2.06	0.04	**1.06**	1.00–1.11
GA care	2.10	0.36	5.79	< 0.001	**8.13**	4.12–17.10
GA care × outness	−0.17	0.05	−3.45	< 0.001	**0.84**	0.76–0.93

The bolding is used to highlight the significant outcomes.

**Table 4:** Negative binomial regression model results for the association between receipt of gender-affirming care and the number of reported negative healthcare experiences, adjusted for sociodemographic characteristics and outness to healthcare providers (*n* = 1383).

	B	SE	*z*	*p*	OR	95%CI
Intercept	0.15	0.15	0.96	0.34	1.16	0.85–1.57
Age	−0.01	0.004	−2.19	0.03	**0.99**	0.98–1.00
Race	0.12	0.08	1.50	0.13	1.13	0.96–1.33
Census region	0.02	0.02	0.99	0.32	1.02	0.98–1.07
Education level	0.18	0.05	3.50	<0.001	**1.20**	1.08–1.33
Outness	0.04	0.01	3.16	0.002	**1.05**	1.01–1.07
GA care	1.12	0.12	9.45	<0.001	**3.09**	2.43–3.93
GA care × outness	−0.09	0.02	−4.72	<0.001	**0.91**	0.88–0.95

The bolding is used to highlight the significant outcomes.

## Data Availability

Underlying data cannot be made publicly available for the following reasons: members of the lesbian, gay, bisexual, transgender, and queer (LGBTQ) communities have experienced significant stigma and discrimination from society including the medical and investigational communities. As such, we are ethically bound to uphold the principle of non-maleficence; we promise our participants to not let any data (including deidentified) fall into the hands of people who may use it to publish stigmatizing results about the LGBTQ communities. As such, we have developed an Ancillary Study process in which investigators interested in using our data submit a brief application that is reviewed by both a Research Advisory Committee (composed of scientists) and a Participant Advisory Committee (composed of participants) to affirm appropriate data use. Details about the Ancillary Study process are available at www.pridestudy.org/collaborate or by contacting us at support@pridestudy.org or 855-421-9991 (toll-free).
